# Role of Redox-Induced Protein Modifications in Spermatozoa in Health and Disease

**DOI:** 10.3390/antiox14060720

**Published:** 2025-06-12

**Authors:** Chika Onochie, Keturah Evi, Cristian O’Flaherty

**Affiliations:** 1 Department of Pharmacology and Therapeutics, McGill University, Montréal, QC H4A 3J1, Canada; chika.onochie@mail.mcgill.ca; 2 Department of Surgery, Urology Division, McGill University, Montréal, QC H4A 3J1, Canada; 3 The Research Institute, McGill University Health Centre, 1001 Decarie Blvd, Montréal, QC H4A 3J1, Canada; 4 Department of Pharmacology, Faculty of Pharmacy, University of Benin, Benin 300283, Nigeria; onome.evi@pharm.uniben.edu; 5 Department of Anatomy and Cell Biology, McGill University, Montréal, QC H4A 3J1, Canada

**Keywords:** male infertility, spermatozoa, redox signaling, oxidative stress, thiol oxidation, post-translational modifications, coenzyme A, antioxidants, protein CoAlation, lipid peroxidation

## Abstract

Male infertility contributes to approximately half of all infertility cases, with most cases associated with oxidative stress. Spermatozoa depend on finely tuned redox signaling for critical processes such as capacitation, motility, and fertilization competence; however, their unique structural and metabolic features render them particularly vulnerable to oxidative damage. Reversible oxidative modifications regulate enzymatic activity, signaling cascades, and structural stability, supporting normal sperm function, whereas irreversible oxidative damage impairs motility, acrosome reaction, and DNA integrity, contributing to male infertility. The intricate balance between physiological redox signaling and pathological oxidative stress demonstrates the potential of redox modifications as biomarkers for infertility diagnosis and as targets for antioxidant-based therapeutic interventions. This review explores the role of redox-induced protein modifications in sperm function, focusing on thiol oxidation, S-nitrosylation, sulfhydration, glutathionylation, CoAlation, and protein carbonylation. By uncovering the mechanisms of these redox modifications, we provide a framework for their modulation in the development of targeted redox interventions to improve male fertility.

## 1. Introduction

Infertility has become a significant global health concern, with a prevalence of 17.5% of infertile couples, and male factors are implicated in roughly half of all cases [1,2]. Oxidative stress, an imbalance in cellular redox status, occurs when the production of reactive oxygen and nitrogen species (RONS) exceeds the capacity of antioxidant defenses and is recognized as a major contributor to male infertility [3]. Several conditions associated with male infertility are linked to oxidative stress, including varicocele (dilation of the pampiniform plexus veins) [4], metabolic diseases such as diabetes [5], cryptorchidism (undescended testes) [6], urogenital infections [7], and exposure to chemotherapeutics such as cyclophosphamide [8]. These conditions may arise from high levels of oxidative stress in the male reproductive tract, leading to impaired sperm development and function. However, a physiological increase in RONS production is sometimes required to ensure normal sperm fertility [9].

**Figure 1:**
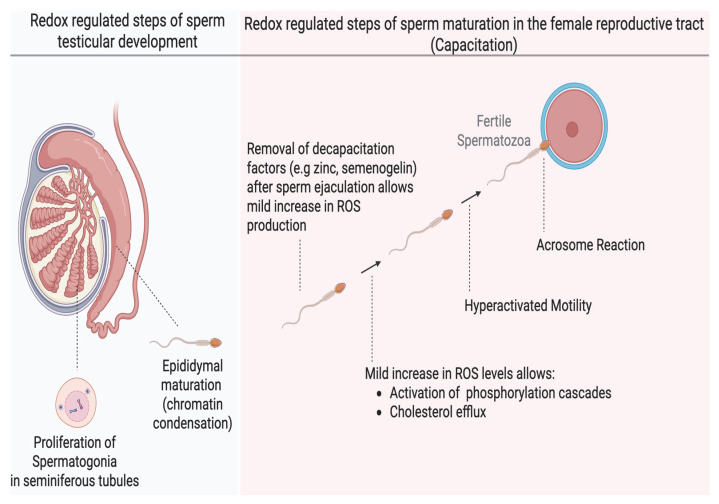
The role of redox regulation in major developmental stages of human spermatozoa. Mild oxidation is important in spermatogonia proliferation during spermatogenesis and chromatin condensation during epididymal maturation in the male reproductive tract. Sperm capacitation is one of the most redox-dependent processes that the spermatozoa must undergo to achieve fertilizing ability and depends on a mild (low) increase in oxidation of certain sperm proteins without altering their motility or viability. Oxidation promotes membrane fluidity and activation of several phosphorylation pathways. These effects endow the sperm with the ability to undergo hyperactivated motility and the acrosome reaction required to successfully fertilize the oocyte. (created in Biorender). https://app.biorender.com/illustrations/683a0eaf546f5abd34903600 (accessed on 3 June 2025).

Human sperm production begins in the seminiferous tubules of the testes, where round spermatogonia undergo multiple stages of differentiation, ultimately giving rise to spermatids [10]. Studies have shown that a mild increase in oxidation is essential for the release of humoral factors that promote proliferation of spermatogonia before differentiation into spermatids [11]. The spermatids then undergo spermiogenesis, characterized by the shedding of excess cytoplasm, DNA compaction, and elongation of the distal centriole to form the flagelum (Figure 1). The immotile spermatozoa are then transported into the epididymis, where they acquire the ability to move and undergo a further nuclear compaction to protect the DNA for the rigorous transit in the female reproductive tract. This chromatin compaction is also regulated by the oxidation of thiol groups into cross-linked disulfide bonds; any dysregulation can compromise the integrity of the sperm DNA [12]. After epididymal maturation, spermatozoa reside in the distal part of the epididymis (cauda epididymis), ready for ejaculation. However, they are still not capable of fertilizing an oocyte, and they acquire this ability in the female reproductive tract, specifically in the Fallopian tubes (oviduct), through a process known as capacitation [13]. Sperm capacitation is an intricately redox-regulated process that endows spermatozoa with functional abilities, including hyperactivated motility and the capability to react to progesterone and undergo the acrosome reaction, an exocytotic event, which are required to fertilize the oocyte [14]. Hyperactivated motility is a specialized motility pattern characterized by the asymmetric movement of the sperm head, accompanied by high-amplitude flagellar movement, which is suited for reaching the oocyte. The acrosome reaction is necessary for the sperm to penetrate the zona pellucida surrounding the oocyte successfully. The changes occurring during capacitation include increased sperm membrane fluidity (through cholesterol efflux), ion influx (especially calcium [Ca^2+^] and bicarbonate [HCO^3−^]), activation of phosphorylation cascades such as the cyclic adenosine monophosphate–protein kinase A (cAMP–PKA) pathway, protein kinase C (PKC), extracellular signal-regulated kinase (ERK), mitogen-activated protein kinase (MEK), and a rise in protein tyrosine phosphorylation [15]. Under physiological conditions, capacitation does not occur within the male reproductive tract and is only induced upon exposure to the unique microenvironment of the female reproductive tract It has now been extensively demonstrated that mild oxidation is required to bring about these necessary changes for capacitation. Cholesterol oxidation precedes its efflux; oxidation is involved in the activation of kinases or inactivation of phosphatases in the signaling cascades of PKA, PKC, ERK, MEK and tyrosine phosphorylation [15,16]. A dysregulation in any of these redox-regulated processes results in impaired capacitation and male infertility.

RONS mediate physiological functions through redox signaling, which is the mechanism by which cells use RONS as second messengers to initiate oxidation-reduction reactions that modulate physiological processes. Redox signaling acts selectively on specific molecular targets, such as amino acid residues in proteins, to induce reversible modifications that alter protein function [17]. Cysteine residues are the primary redox targets due to their highly nucleophilic thiol (–SH) groups, which are easily oxidized to form reversible intermediates [18]. Through these oxidative modifications, RONS (as well as reactive nitrogen and sulfur species) modulate the activity, interactions, and localization of proteins, thereby controlling essential sperm processes from spermatogenesis [19], through capacitation [20] to fertilization [21]. While cysteine thiols are the most common redox-sensitive sites, evidence indicates that other residues can also be redox-regulated. Methionine residues, for instance, can be oxidized to methionine sulfoxide (a modification that can be enzymatically reversed) as part of cellular signaling [22]. Moreover, redox signaling may target non-protein molecules; for example, oxidants can modify cholesterol in sperm membranes, influencing membrane fluidity and signaling events [16]. The key feature of redox signaling is that these modifications are reversible. Notable reversible oxidative modifications regulating sperm function include thiol sulfenylation (formation of cysteine–SOH), S-nitrosylation (attachment of an NO group to a cysteine thiol), S-glutathionylation (forming a mixed disulfide between protein thiol and glutathione), S-CoAlation (forming a mixed disulfide between protein thiol and coenzyme A), persulfidation (also called sulfhydration, adding an –SSH moiety to a thiol), and methionine oxidation to sulfoxide. By transiently switching protein thiols between reduced and oxidized states, or toggling other redox-sensitive residues, spermatozoa leverage redox signaling to regulate phosphorylation cascades, enzyme activities, and structural protein interactions critical for normal sperm function.

Oxidative stress arises when ROS levels exceed the physiological threshold and antioxidant mechanisms are overwhelmed [23]. In this state, the beneficial redox signaling is overwhelmed by excessive, non-specific oxidation of biomolecules, leading to cellular injury rather than regulation. Spermatozoa are particularly vulnerable to oxidative stress for several reasons: their plasma membranes are enriched in polyunsaturated fatty acids that are prone to ROS-driven peroxidation [24], their cytoplasm contains minimal antioxidant enzymes (as sperm lose most of their cytoplasm during maturation) [25], and after ejaculation they are no longer bathed in the protective antioxidant-rich seminal plasma [19,26]. Consequently, unchecked ROS can inflict widespread, irreversible damage on sperm components. Proteins exposed to high ROS may acquire irreversible oxidative modifications; for example, cysteine thiols can be hyperoxidized beyond sulfenic to sulfinic and sulfonic acids that inactivate the protein [27]. Protein tyrosine residues can be oxidatively modified to 3-nitrotyrosine (protein tyrosine nitration), a stable marker of peroxynitrite damage [28]. Even usually reversible modifications can become pathologically persistent under severe oxidative stress: for instance, excessive ROS can lead to sustained S-glutathionylation of proteins that are not readily reversed [29]. One of the most detrimental consequences of oxidative stress in sperm is lipid peroxidation, a chain reaction that not only damages the sperm membrane (affecting fluidity and integrity) but also generates aldehydes (like 4-hydroxynonenal (4-HNE) and malondialdehyde) that form adducts with proteins and DNA [30]. In addition, reactive carbonyl species generated during lipid peroxidation can react with proteins, causing protein carbonylation (an irreversible modification that compromises protein function) [31]. In addition to generating aldehydes, oxidative stress may also directly oxidize specific bases of DNA, such as guanosine bases to form 8-oxo-2′-deoxyguanosine (8-oxo-dG) [32]. Collectively, these non-selective oxidative injuries impair sperm motility, viability, DNA integrity, and the ability to fertilize an oocyte, thereby contributing to male infertility.

**Figure 2:**
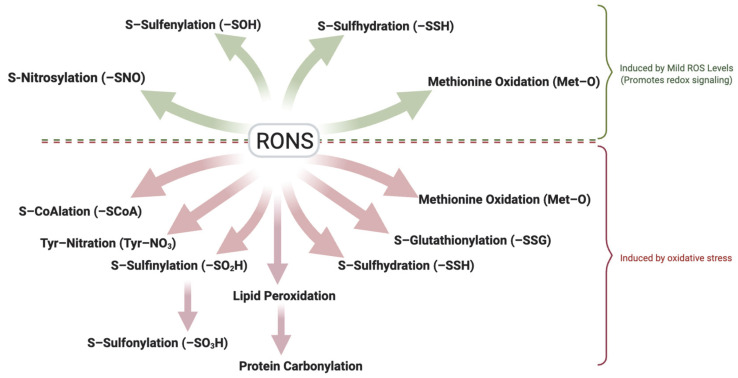
Dual Role of Reactive Oxygen and Nitrogen Species (RONS) in Sperm Function. Controlled, mild (low) levels of RONS act as essential signaling mediators that promote sperm function through reversible oxidative modifications, including sulfenylation (–SOH), S-nitrosylation (–SNO), S-sulfhydration (–SSH), and methionine oxidation (-MetO). These reversible modifications regulate motility, capacitation, acrosome reaction, and zona pellucida binding. In contrast, elevated RONS levels lead to oxidative stress, resulting in irreversible modifications such as protein carbonylation through lipid peroxidation and tyrosine nitration. Persistent oxidation can convert typically reversible modifications like S-glutathionylation, S-CoAlation, and S-sulfinylation into signals of damage. Oxidative stress and its associated modifications disrupt membrane integrity, cytoskeletal function, and DNA stability, ultimately impairing fertilization competence and contributing to male infertility. The ability of spermatozoa to balance redox signaling and oxidative damage is critical for preserving reproductive potential. Created in BioRender. https://app.biorender.com/illustrations/681527a353b88e93b9976da4, accessed 3 June 2025.

It is important to distinguish between physiological ROS levels (mild oxidation) and pathological oxidative stress. Although precise in vivo quantification remains challenging due to methodological limitations [33], the detection of specific oxidative modifications can serve as indicators of RONS levels. Reversible modifications such as S-sulfenylation, S-nitrosylation, S-sulfhydration and methionine oxidation typically reflect mild oxidation, while irreversible modifications including sulfonic acid formation, tyrosine nitration, and protein carbonylation are hallmarks of oxidative stress. Also, modifications such as S-glutathionylation, S-CoAlation and S-sulfinylation which are usually reversible under physiological conditions, may represent oxidative stress if oxidative conditions persist (Figure 2). Understanding the spectrum of redox modifications in spermatozoa holds significant therapeutic promise. Specific oxidative modifications may serve as sensitive biomarkers of sperm quality, distinguishing healthy redox-regulated sperm function from pathological oxidative stress. For example, assays of thiol oxidation status [34] or detection of protein adducts (like 4-HNE or carbonylated proteins) in sperm might indicate oxidative damaging conditions associated with male infertility [35]. Moreover, this knowledge opens avenues for targeted antioxidant therapies that aim to mitigate harmful oxidative damage while sparing or even bolstering the beneficial redox signaling required for fertility [36]. A deeper understanding of these modifications will support the development of diagnostic tools and targeted treatments to improve male fertility outcomes. This review explores the major redox-induced protein modifications reported in spermatozoa, their molecular targets, and their impact on key functional processes such as motility, capacitation, the acrosome reaction, zona pellucida binding, and genomic integrity. We place particular emphasis on sperm capacitation, given its high sensitivity to redox regulation.

## 2. Thiol Oxidation (Sulfenic, Sulfinic and Sulfonic Acid)

**Figure 3:**
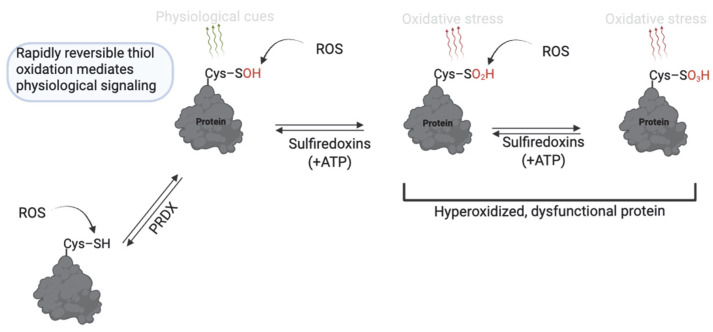
Thiol oxidation to sulfenic, sulfinic and sulfonic acid in sperm function. Cysteine thiols undergo reversible oxidation to sulfenic acid (–SOH) under mild ROS conditions, enabling redox signaling. With sustained oxidative stress, further oxidation to sulfinic (–SO_2_H) or sulfonic (–SO_3_H) acids occurs; these forms are largely irreversible or only reversible through energy-dependent mechanisms and are associated with pathological oxidative stress. Created in BioRender. https://app.biorender.com/illustrations/681527a353b88e93b9976da4, accessed 3 June 2025.

RONS levels are essential for normal sperm function, whereas excessive RONS levels cause oxidative damage and infertility [9,24]. ROS can act by stepwise oxidation of protein cysteine thiols (–SH) to form modifications that reflect cell function or pathology [37]. The reaction of protein thiols with ROS, such as O_2_^−^ and H_2_O_2_, at mild levels initially yields cysteine sulfenic acid (R–SOH), which is involved in reversible redox signaling for sperm function (Figure 3). Sulfenic acid can either reverse to free thiols (via cellular antioxidants) or form a disulfide bond with another cysteine. Sustained oxidation, or at high levels, will promote further oxidation of sulfenic acid to sulfinic acid (R–SO_2_H), or under severe oxidative stress, to sulfonic acid (R–SO_3_H) [27]. Sulfinic and sulfonic acids are associated with pathology since they are not rapidly reversible and indicate oxidation beyond normal signaling ranges or oxidative stress. Although energy-dependent mechanisms can reverse sulfinic acid, the energy demand renders it unsuitable for the rapidly reversible nature of redox signaling [38]. Sulfonic acid is generally considered irreversible in vivo, except for certain peroxiredoxins, where sulfiredoxin or sestrins can reverse hyperoxidized Cys–SO_3_H [39,40]. The presence of these modifications significantly regulates sperm physiology.

Sperm capacitation begins with the removal of decapacitation factors such as zinc and semenogelin, which normally keep spermatozoa in a quiescent, non-capacitated state [41]. The removal of these factors relieves inhibition on a yet-to-be-identified sperm oxidase, leading to an uninhibited increase in RONS production [9]. These RONS then act as second messengers, initiating redox signaling through reversible thiol oxidation, particularly the formation of sulfenic acid (Cys–SOH) on target proteins [9]. Sulfenic acid formation targets protein phosphatases and kinases to ensure the phosphorylation events of capacitation [42,43,44]. Many protein tyrosine phosphatases (PTPs) have an active-site cysteine that is highly susceptible to oxidation [45,46]. Oxidation to sulfenic acid inactivates these phosphatases transiently, allowing an accumulation of phosphorylated proteins [20,47]. In parallel, RONS promote activation of kinases such as PKC, ERK, and PI3K, in part by stimulating cAMP production via soluble adenylyl cyclase (sAC), which activates the PKA pathway [48]. These phosphorylation events are essential for promoting the hyperactivation and acrosome reaction, hallmarks of sperm capacitation. Beyond phosphorylation, redox-sensitive thiols on sperm-egg binding proteins (e.g., IZUMO4, SPAM1) and acrosomal proteins (e.g., ACRBP, ZPBP1) are regulated through reversible thiol oxidation, indicating that thiol modifications fine-tune the sperm’s ability to bind and penetrate the oocyte [49].

The increase in RONS production during capacitation may induce oxidative stress if not regulated. Actin and tubulin, key structural elements of the sperm flagellum, are known targets of uncontrolled oxidative stress. Their oxidation impairs cytoskeletal integrity, leading to abnormal flagellar motility [50]. CatSper channels have redox-sensitive thiol groups and, when oxidized, impair the influx of calcium required for hyperactivation and fertilization [51]. Similarly, metabolic enzymes such as GAPDHS, LDH-C4, citrate synthase, and aconitase are susceptible to oxidative inactivation [52]. GAPDHS, which fuels flagellar glycolysis, contains an oxidation-sensitive cysteine that becomes inactivated by sulfinic oxidation during oxidative stress [53]. This redox-sensitive loss of function correlates with decreased ATP production, poor motility, and infertility in humans.

Peroxiredoxins (PRDXs) serve as the major ROS scavengers that prevent unregulated rise in ROS levels during capacitation [54]. These thiol-based enzymes are abundant in human spermatozoa and catalyze the reversible formation of sulfenic acid at their active-site cysteine residues, effectively scavenging H_2_O_2_ and other peroxides [55]. PRDXs are then regenerated via the thioredoxin system, allowing continuous cycling and preventing ROS accumulation. When PRDXs become hyperoxidized to sulfinic acid, however, their activity is compromised, leading to unchecked oxidative stress. The importance of peroxiredoxins and the reversibility of their thiol oxidation in controlling ROS levels during capacitation is underscored by several lines of evidence in human infertility and experimental models. In cases of idiopathic male infertility, reduced expression and increased hyperoxidation of PRDX1 and PRDX6 are associated with impaired motility and elevated lipid peroxidation [35,54]. Experimental inhibition of PRDXs in bull sperm similarly results in increased oxidation of flagellar and metabolic proteins and decreased motility [30]. Mouse models lacking PRDX6 show elevated oxidative stress, reduced motility, and infertility phenotypes reversible with antioxidant diets [36].

**Figure 4:**
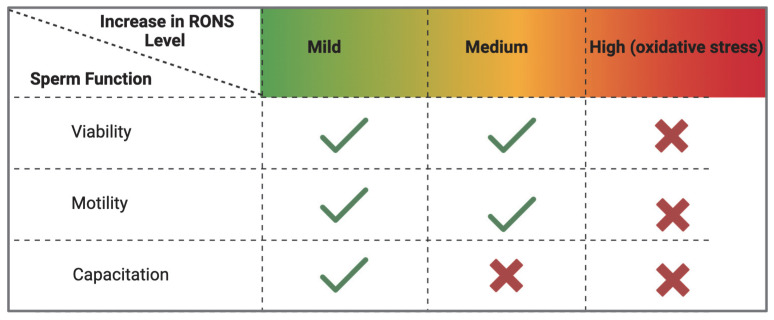
Effect of oxidation levels on sperm function during capacitation. An increase in RONS levels is necessary to initiate sperm capacitation. These levels are kept within mild (low) levels by peroxiredoxins whose catalytic cysteines undergo reversible oxidation to sulfenic acids. When peroxiredoxins are inhibited, there is an increase in RONS levels that impairs sperm capacitation. Absence of peroxiredoxins renders sperm incapable of controlling RONS levels, leading to non-specific oxidative stress, leading to impaired sperm viability, motility, and capacitation. (created in BioRender). https://app.biorender.com/illustrations/683a1635172c8b1ee82a83a6 (accessed on 3 June 2025).

In summary, there is an optimal window of mild oxidation for successful sperm capacitation (Figure 4). Within that window, sulfenic acid formation represents a necessary form of redox signaling that activates processes such as protein phosphorylation and hyperactivation. Beyond that window, higher-order oxidation states like sulfinic acid become detrimental. Peroxiredoxins enforce this window by scavenging ROS and maintaining them at low levels. If PRDXs are impaired during oxidative stress (hyperoxidized to sulfinic acid), capacitation is impaired without altering sperm motility or viability. In infertility conditions where PRDXs are defective (as in knockout animal models), there is impairment of sperm viability, motility and function.

## 3. S-Nitrosylation and Tyrosine Nitration

**Figure 5:**
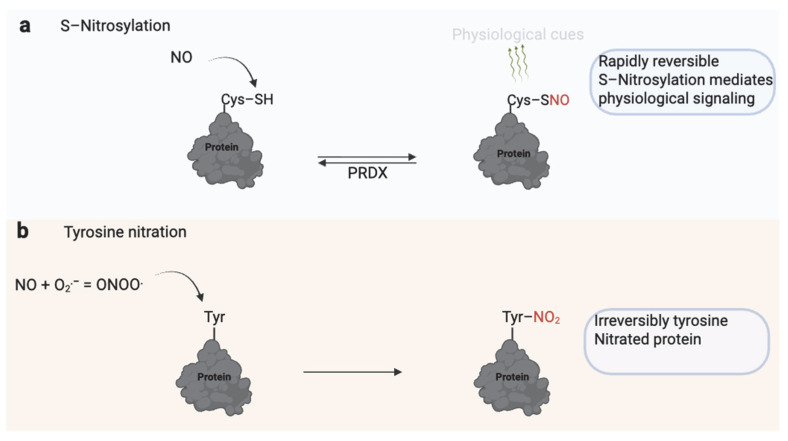
Dual Role of Nitric Oxide (NO) in Sperm Function. (**a**) S-nitrosylation involves the reversible addition of an NO group to the thiol (–SH) group of reactive cysteine residues, forming S-nitrosothiols (–SNO). This modification regulates protein activity, signaling, and localization and is rapidly reversible through enzymatic systems such as peroxiredoxins (PRDX). S-nitrosylation modulates physiological processes, including motility, capacitation, and the acrosome reaction. (**b**) Excessive or sustained NO reacts with superoxide (O_2_^−^) to form peroxynitrite (ONOO^−^), which irreversibly modifies tyrosine residues through nitration. Tyrosine nitration alters protein structure and function, often leading to impaired signaling, mitochondrial dysfunction, and reduced sperm motility. While S-nitrosylation serves as a physiological redox signal, tyrosine nitration is typically associated with nitrosative stress and male infertility. Created in BioRender. https://app.biorender.com/illustrations/681527a353b88e93b9976da4, accessed 3 June 2025.

Since its discovery as a mediator of endothelium-dependent relaxation, nitric oxide (NO) has emerged as a key physiological signaling molecule in various biological systems [56]. One of its primary regulatory mechanisms is S-nitrosylation, a reversible modification of protein thiol groups that alters protein activity, stability, or localization [56] (Figure 5). Low, tightly regulated NO levels in spermatozoa promote essential fertilization processes through S-nitrosylation. These include enhanced motility [57], capacitation [58], and the acrosome reaction [59].

Emerging evidence supports a role of NO through nitrosylation in sperm cryosurvival. Mild NO exposure has been shown to activate protective stress response pathways involving heat shock proteins, enhancing post-thaw motility and fertility in human spermatozoa [60]. A proteomic study of human sperm exposed to sublethal NO levels before cryopreservation revealed that NO enhances redox homeostasis by activating thioredoxins, key proteins involved in protecting against the oxidative effects of cryocapacitation [61]. Furthermore, while cryopreservation typically increases oxidative phosphorylation (OXPHOS) proteins, especially from mitochondrial complex I, raising ROS levels, NO pre-treatment reduced this effect, decreasing the number of upregulated complex I proteins [62].

NO is continuously produced during sperm capacitation, promoting key phosphorylation events [9]. Recently, it was suggested that NO may further enhance phosphorylation by activating cGMP, which competes with cAMP for degradation by phosphodiesterase [63]. This competition indirectly sustains cAMP levels and supports PKA signaling, thereby contributing to the regulation of capacitation and the acrosome reaction. Additionally, NO-mediated S-nitrosylation of ryanodine receptors (RyRs) facilitates intracellular Ca^2+^ release, promoting hyperactivation and motility in porcine spermatozoa [64]. Notably, protein S-nitrosylation also increases during capacitation and often occurs on the same cysteine residues susceptible to sulfenic formation. Therefore, both modifications can modulate enzyme activity and protein–protein interactions in tandem [65].

New strategies for NO delivery to the sperm are also under exploration. For example, citrate has been identified as an endogenous source of NO that sustains redox signaling during human sperm capacitation [66], and S-nitroso-N-acetylpenicillamine (SNAP), a NO donor, prolongs the fertilization potential of rooster sperm before artificial insemination [67]. Similarly, pentoxifylline, a phosphodiesterase inhibitor, was shown to improve NO production in rams following oral administration, which correlated with increased sperm tolerance to heat stress [68]. Similarly, caffeine has been shown to stimulate NO production in human spermatozoa correlated with improved sperm motility parameters [69].

Notably, the beneficial role of NO hinges on concentration and context. When NO levels become excessive or persist, it reacts with superoxide to form peroxynitrite (ONOO^−^). Unlike S-nitrosylation, ONOO^−^ drives tyrosine nitration, an irreversible post-translational modification that disrupts protein function and is linked to oxidative stress due to inhibition of PRDX6 [70] (Table 1). Under pathological conditions or in Prdx6^−/−^-deficient mouse models, ONOO^−^ levels rise unchecked, leading to oxidative damage [71]. Peroxynitrite may interfere with normal cell signaling pathways. It disrupts PKA substrate phosphorylation and GSK-3α activity, overriding normal regulation in porcine spermatozoa [55,72]. While ONOO^−^ is typically considered harmful, regulated tyrosine nitration may have physiological roles. Kalezic et al. (2018) found that seminal plasma from infertile men had lower levels of 3-nitrotyrosine compared to normospermic infertile men, and this decrease correlated with poorer sperm motility, morphology, and count, suggesting that controlled nitration might support sperm function and deserves further investigation [73].

The ultimate consequence of ONOO^−^ in spermatozoa is mitochondrial permeability transition (MPT)-driven necrosis, a form of cell death characterized by mitochondrial membrane collapse and energy failure [77]. This process is closely linked to impaired ATP production because ONOO^−^ disrupts oxidative phosphorylation by damaging mitochondrial complex proteins, leading to an energy crisis within the sperm cell and compromising its motility and survival [74]. Furthermore, ONOO^−^ triggers extensive lipid peroxidation, especially in the sperm plasma membrane rich in polyunsaturated fatty acids, resulting in loss of membrane fluidity and integrity, essential for motility, capacitation, and fusion with the oocyte. DNA oxidation is another consequence of ONOO exposure, leading to strand breaks and base modifications that reduce the genetic quality of sperm and may impact fertilization outcomes and embryo development [76]. These effects collectively reduce sperm motility and viability, impairing overall fertilizing capacity. In an experimental model combining NO and hydrogen sulfide (H_2_S) exposure, high concentrations of sodium nitroprusside (SNP), a NO donor, destabilized the acrosome membrane, a process attributed to ONOO^−^-mediated oxidative damage [57].

Given the relative stability and reactivity of peroxynitrite, therapeutic strategies targeting its neutralization are being explored. One such compound, FeTPPS (5,10,15,20-tetrakis(4-sulfonatophenyl)porphyrinate iron(III) chloride), has been shown to mitigate the harmful effects of peroxynitrite on human spermatozoa [78]. Treatment with FeTPPS preserved ATP levels, mitochondrial membrane potential, DNA integrity, motility, and viability under experimentally induced nitrosative stress conditions, making it a promising therapeutic agent for oxidative stress-induced infertility. Another study using Prdx6^−/−^ mice, an established model of oxidative-stress-induced subfertility, demonstrated that dietary supplementation with a γ-tocopherol-rich mixture and ascorbic acid significantly restored sperm quality and fertility [36]. Reduced levels of lipid peroxidation and DNA oxidation, and markedly decreased tyrosine nitration levels, particularly in the acrosomal and flagellar regions, were observed in mice fed with this vitamin mixture.

## 4. Protein S-Glutathionylation

Glutathionylation is a reversible oxidative post-translational modification that involves the reversible covalent attachment of glutathione, a tripeptide thiol, to reactive cysteine residues on target proteins. By forming disulfide bonds with protein thiols, glutathionylation protects cysteine residues from hyperoxidation, modulates protein structure, and participates in functional redox signaling.

Protein glutathionylation is catalyzed principally by the glutathione-S-transferases (GSTs) [79]. Glutaredoxins (typically known for deglutathionylation) may also facilitate glutathionylation under oxidative stress conditions [80]. Other enzymes, such as the glyoxalases, also catalyze protein glutathionylation [81]. The ROS scavenger function of glutathione is mediated by glutathione peroxidases that neutralize ROS, using reduced GSH as a cofactor, resulting in oxidized glutathione GSSG [82]. Oxidized GSSG is reduced back to regenerate GSH by glutathione reductase. In somatic cells, the redox regulatory role of protein glutathionylation contributes to normal cell physiology. Protein glutathionylation affects diverse processes such as gene transcription through heat shock proteins [83], cellular signaling through calcium homeostasis [84], or phosphorylation cascades [85]. On the other hand, it contributes to disease pathophysiology during prolonged oxidative stress as reversibility of the reaction is compromised [86].

Research has shown that proteins in spermatozoa from human [29], bull [65], mouse [87], and other species undergo glutathionylation. GSTs are mainly associated with the sperm plasma membrane and are involved in detoxification [88], inhibiting sperm motility and capacitation by affecting MAPK signaling cascades [20] and their requirement for successful oocyte recognition by binding to ZP4 [89]. Polymorphisms in GSTM1 and GSTT1, particularly the null genotypes, are associated with reduced sperm motility and increased oxidative stress, highlighting the protective role of these enzymes in male fertility [90]. GSTs are also a positive marker of fertilization success and cryotolerance [91]. Although GSTs are essential to maintain sperm quality, very high levels correlate with low sperm quality and litter sizes in pigs and bulls [92]. Glutathione peroxidases (GPXs) are selenocysteine antioxidant enzymes that reduce hydrogen peroxide and lipid hydroperoxides using glutathione (GSH) as a cofactor. In mature spermatozoa, GPX4 is the major isoform, primarily in the mitochondria (mGPX4), constituting over 50% of the mitochondrial sheath. GPX4 provides structural integrity to the sperm midpiece but lacks enzymatic antioxidant activity [93]. A nuclear isoform (nGPX4) also exists; however, it appears to play a limited role in antioxidant defense, as mice lacking nGPX4 remain fertile [94].

Despite its reversible nature, S-glutathionylation is inhibitory to sperm capacitation. Studies in bull sperm have shown that capacitation is associated with dynamic changes in glutathionylation, with proteins becoming increasingly deglutathionylated [95]. Key proteins that undergo deglutathionylation in these studies include IZUMO4, which is involved in sperm–oocyte fusion and shows deglutathionylation during capacitation [65]. Similarly, ODF2, a structural protein of the sperm flagellum essential for hyperactivation, and TEKT2, which contributes to the organization of flagellar motility, undergo deglutathionylation. If the cellular redox balance tips too far towards oxidation during capacitation, excessive S-glutathionylation can inactivate critical enzymes and block capacitation [29]. Peroxiredoxins also contribute to governing this balance of glutathionylation during sperm capacitation [96]. In bull sperm, inhibition of PRDXs caused an abnormal increase in protein S-glutathionylation and a corresponding decrease in capacitation-associated tyrosine phosphorylation [96]. Specifically, PRDX inhibition led to the glutathionylation of GAPDHS and AKAP proteins’ dephosphorylation. Similarly, in human sperm, pharmacological blockade of PRDX enzymes prevented the normal increase in protein phosphorylation during capacitation [97,98]. The mechanism is that without active PRDXs, ROS levels increase and drive excessive S-glutathionylation, which inactivates essential signaling pathways such as PKA and PKC signaling.

Glutathionylation also regulates sperm function through molecular chaperones involved in protein homeostasis [99]. Hsp70 is a chaperone essential for protein folding, motility, and capacitation, while also acting as an anti-apoptotic factor supporting sperm viability. Its activity is tightly regulated by redox mechanisms [100]. Glutathionylation of HspA1A (hHsp70) at Cys-574 and Cys-603 causes unfolding of the α-helical lid [101]. This change allows intramolecular binding to the substrate-binding site, mimicking substrate interaction and activating ATPase activity. However, it also prevents normal substrate binding, such as with heat shock transcription factor 1 (Hsf1), impairing Hsp70′s role in protein refolding [101]. Since spermatozoa lack transcriptional activity, glutathionylation of Hsp70 may be a reversible mechanism to modulate chaperone activity during sperm maturation and transit through the reproductive tract [100]. Proteins susceptible to other oxidative modifications, such as S-nitrosylation, are often also targeted by S-glutathionylation, sometimes at the same cysteine residues [65]. This overlap points to a coordinated and dynamic redox network in spermatozoa, where modifications may occur sequentially or competitively to regulate protein function. For instance, S-nitrosylation can prime specific thiols for subsequent S-glutathionylation, as demonstrated for GAPDH, linking nitrosative and oxidative signaling pathways [102].

The antioxidant effect of glutathionylation in semen cryopreservation has been the topic of recent research, with studies showing that supplementing glutathione in the cryopreservation media improves semen quality in human [103], goat [104], rooster [105], bull [106], and fish [107]. These effects are attributed to reduced lipid peroxidation [108], ROS scavenging [109], and promoting sperm nuclear decondensation post-fertilization [110]. Optimal levels of GSH improved GPX-like activity, reduced apoptotic-like changes, and DNA methylation in rooster sperm [111]. Cryopreserved rooster sperm can also utilize glutathione precursor, cysteine, for the same effect [112]. This is supported by findings that active protein translation of cystathionine β-synthase (CBS) and cystathionine γ-lyase (CTH) involved in glutathione biosynthesis are active during sperm incubation [113]. Through its antioxidant function, glutathione is known to promote semen efficiency during cryopreservation [114]. Supplementing sperm with cysteine prevents oxidative damage, suggesting that sperm utilize exogenous cysteine to replenish GSH and maintain redox balance [115].

Clinical studies have reported dysregulation of the oxidoreductases in infertile men, correlating with reduced sperm concentration, motility, and morphology [116,117,118]. Among these, thioredoxin/glutathione reductase (TXNRD3) stands out due to its unique structure, comprising both a thioredoxin reductase module and an additional glutaredoxin domain. This dual configuration enables TXNRD3 to reduce both thioredoxin and glutathione. TXNRD3, which is highly expressed in the testes, has been implicated in the redox signaling during fertilization [119]. It has been demonstrated that TXNRD3 deficiency in mice leads to disrupted thiol redox balance and impaired epididymal maturation, contributing to reduced sperm motility and fertilization capacity [87]. Proteomic analyses from the same study revealed a broad range of substrates reduced by TXNRD3 during sperm maturation, suggesting its involvement in sperm quality control. ROS induce a dose-dependent increase in glutathionylation, which, under oxidative stress conditions, disrupts critical sperm processes, impairing motility and capacitation [29].

Modifications of metabolic enzymes impair glycolysis and oxidative phosphorylation, leading to decreased ATP production and compromised motility. S-glutathionylation of GAPDH at its catalytic cysteine residue (Cys152) significantly inactivates the enzyme, leading to a loss of its glycolytic function [102]. This modification reduces the thermal stability of GAPDH, making it more prone to unfolding and degradation. Similarly, tubulin, a major component of the sperm flagellum, is particularly susceptible to S-glutathionylation, which can disrupt flagellar function and, consequently, sperm motility [54]. These disruptions are particularly pronounced in asthenozoospermic men, where increased oxidative stress is associated with elevated glutathionylation and sperm dysfunction [120].

The role of PRDXs, particularly PRDX6, is crucial in mitigating oxidative damage in spermatozoa. The absence of PRDX6 has been shown to exacerbate oxidative stress, leading to increased S-glutathionylation of sperm proteins, ultimately resulting in decreased motility and fertilization capability [121]. In PRDX6-deficient spermatozoa, excessive glutathionylation is accompanied by increased oxidative stress markers, such as lipid peroxidation, impairing their function. In the context of cryopreservation, it has been proposed that targeted antioxidant delivery to mitochondria may help restore redox balance, prevent excessive glutathionylation, and improve sperm quality during assisted reproduction [122].

## 5. Protein S-CoAlation

Coenzyme A (CoASH) is a vital and versatile cofactor in all living cells [123]. It is derived from pantothenic acid, ATP, and cysteine in a universally conserved biosynthetic pathway [124]. CoASH is involved in many cellular functions, including catabolic processes such as the β-oxidation of fatty acids and anabolic pathways like fatty acid elongation for triglyceride biosynthesis [125]. Additionally, CoASH plays key roles in metabolic regulation, gene expression, and detoxification [125,126]. The functional diversity of CoASH is largely due to its reactive thiol group, which enables the formation of high-energy thioester bonds with intermediates [125].

**Figure 6:**
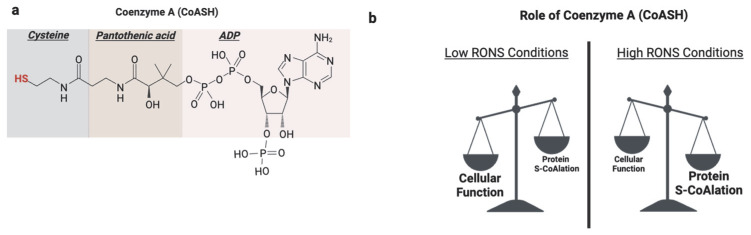
Role of protein S-CoAlation. (**a**) Coenzyme A (CoASH) is composed of adenosine diphosphate (ADP), pantothenic acid, and cysteine. Its reactive thiol group on the cysteine moiety underlies its classical metabolic roles and function in redox regulation through protein CoAlation. (**b**) Under basal physiological conditions, CoASH primarily functions as a metabolic cofactor in fatty acid metabolism and the TCA cycle processes. However, under oxidative stress, CoASH is increasingly diverted toward antioxidant defense by forming mixed disulfides with protein cysteine residues, a reversible modification known as protein CoAlation. Created in BioRender. https://app.biorender.com/illustrations/681527a353b88e93b9976da4, accessed 3 June 2025.

Beyond these classical roles, a newly recognized function of the CoASH thiol is its participation in redox signaling and antioxidant defense through a novel post-translational modification known as protein CoAlation (Figure 6). Protein CoAlation is a reversible, enzyme-mediated modification in which the thiol group of CoASH forms mixed disulfides with the cysteine thiol groups of proteins [127]. It closely resembles glutathionylation in several ways. Both modifications target reactive cysteine residues, are induced under oxidative stress, and function to protect proteins from hyperoxidation. These modifications are reversible and tightly regulated; CoAlation is reversed by thioredoxins and potentially by yet-unidentified CoA-specific reductases [128]. CoAlation, similarly to glutathionylation, can influence protein activity, structure, and interactions, thereby modulating redox signaling and metabolic processes.

Protein CoAlation is an important redox-regulatory mechanism, as demonstrated in the modulation of ribosomal protein S6 kinase 1 (S6K1), a key effector of the mTOR/PI3K pathway [129]. Under oxidative stress, cells may induce CoAlation at Cys217 within the S6K1 activation loop instead of leading to protein degradation or irreversible oxidation. This modification inhibits S6K1 activity independently of phosphatase action, preserving the protein in a reversible, inactive form [129]. This example illustrates how CoAlation can function as a protective switch, coupling redox signals to regulate critical signaling proteins.

CoAlation may offer a higher regulatory potential than glutathionylation despite its similarity to glutathionylation. The unique properties of CoASH, including its bulkier and more complex structure, introduce steric and electrostatic bonds that can influence protein folding, stability, localization, and interaction networks in ways that glutathione cannot. For example, the diphosphate groups in the CoASH ADP moiety contribute to inhibiting the catalytic activity of NME1/NME2 during CoAlation [130,131], demonstrating that CoAlation can exert direct regulatory effects beyond thiol modification. Furthermore, unlike glutathionylation, which readily modifies broadly accessible thiols, CoAlation may demonstrate greater specificity by accessing cysteine residues within defined structural or electrochemical environments. This specificity is supported by mechanistic work from Tossounian et al. (2022), who showed that CoAlation engages protein targets through distinct binding modes driven by structural recognition [132]. For example, the ADP moiety of CoASH may bind to hydrophobic pockets in nucleotide-binding domains such as the Rossmann fold, positioning the thiol near otherwise inaccessible cysteines. This targeted mechanism enables precise modification, as seen in the CoAlation-dependent inhibition of Aurora kinase A [133].

**Figure 7:**
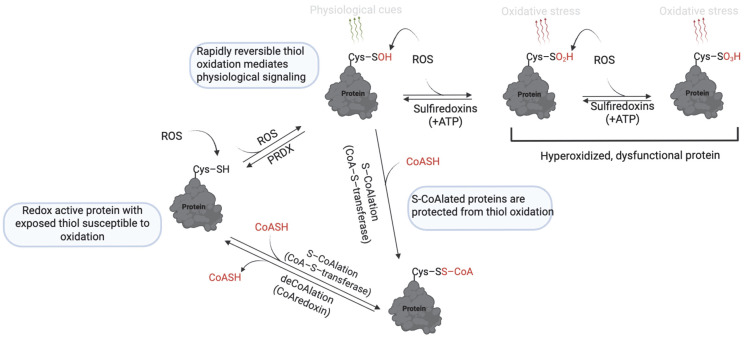
Mechanism of protein S-CoAlation. At low levels of ROS, thiol oxidation to sulfenic acid (–SOH) is reversible and mediates redox signaling. However, further oxidation to sulfinic (–SO_2_H) and sulfonic (–SO₃H) acids is irreversible and leads to loss of protein function. S-CoAlation acts as a protective checkpoint by capping the thiol as a mixed disulfide with CoASH, thereby shielding it from irreversible oxidation. This modification is reversible, allowing proteins to be restored once redox homeostasis is reestablished. Thus, S-CoAlation preserves protein function under oxidative stress and supports cellular redox balance. https://app.biorender.com/illustrations/681527a353b88e93b9976da4, assessed on 3 June 2025.

During oxidative stress conditions, the function of CoASH shifts from metabolic and redox regulation to antioxidant function [134]. The molecular mechanisms are proposed to be analogous to glutathionylation involving a CoA-S-transferase to catalyze the forward reaction, an unknown CoA-redoxin to catalyze deCoAlation, CoA-peroxidase to scavenge ROS, and CoA-disulfide reductase to reduce oxidized CoASSCoA [134] (Figure 7). Increased protein CoAlation has been observed in diverse systems, including mammalian cells [127], prokaryotes, including bacteria [135], and amoeba [136] after exposure to oxidants (Table 2). This modification affects a broad range of proteins, including metabolic enzymes [137], transcription factors [138], antioxidant enzymes [139], cell cycle regulators [133], and signaling kinases [129].

Notably, the antioxidant function of CoAlation allows for the protection and full functional recovery of sensitive proteins. For example, during oxidative stress in Staphylococcus aureus, CoAlation transiently inactivates GAPDH, protecting its cysteines from hyperoxidation, with complete reactivation upon removing the oxidant [120]. A similar reversible effect has been reported for peroxiredoxin 5, a key ROS-scavenging enzyme that relies on reversible cysteine oxidation for its activity [139].

Given the ubiquity and regulatory potential of protein CoAlation, its role in spermatozoa warrants investigation. Human spermatozoa are particularly susceptible to oxidative stress due to their high content of polyunsaturated fatty acids (PUFAs), limited cytoplasmic antioxidant capacity, and relatively low levels of intracellular glutathione. Meanwhile, redox signaling is essential for regulating key functional processes such as capacitation, motility, and the acrosome reaction. In this context, CoAlation may act as a complementary redox-regulatory mechanism, protecting proteins from oxidative damage while modulating signaling pathways required for fertilization. Recent findings show that human spermatozoa exhibit increased CoAlation under oxidative stress, suggesting a potential antioxidant role, possibly involving peroxiredoxins, which are major ROS scavengers in spermatozoa [140]. The same study also showed that basal CoAlation levels were significantly reduced before the phosphorylation-dependent events of capacitation. This reduction may reflect a regulatory mechanism by which spermatozoa relieve CoAlation-mediated inhibition of key kinases, allowing phosphorylation to proceed and initiating capacitation.

## 6. Protein Carbonylation and Lipid Peroxidation

Protein carbonylation is a non-enzymatic and irreversible oxidative modification that introduces carbonyl (–CO) moieties into amino acid side chains [141]. It primarily affects lysine (Lys), arginine (Arg), proline (Pro) and threonine (Thr) residues [142]. Carbonylation can occur through direct oxidation of protein side chains by ROS (primary carbonylation) or indirectly via Michael addition and Schiff-base reactions with reactive aldehydes generated during lipid peroxidation (secondary carbonylation) [143]. The latter pathway links lipid peroxidation to protein carbonylation, as aldehydes generated by membrane lipid peroxides diffuse into the cytosol and covalently modify proteins [144]. Lipid peroxidation is initiated when ROS, particularly hydroxyl radicals, abstract hydrogen atoms from polyunsaturated fatty acid (PUFA) chains in sperm membranes. The resulting lipid radicals rapidly react with oxygen to form lipid peroxyl radicals, perpetuating a damaging chain reaction. Unless terminated by antioxidants such as vitamin E, lipid hydroperoxides accumulate, leading to cytotoxic byproducts, including malondialdehyde (MDA) and 4-hydroxynonenal (4-HNE).

Protein carbonylation accumulates in cells under oxidative stress or aging, marking proteins for misfolding, dysfunction, and degradation [145,146]. Notably, intrinsic sperm quality influences basal protein carbonylation levels; comparative studies in bull sperm have shown that low-quality ejaculates exhibit higher carbonylation, particularly targeting mitochondrial and cytoskeletal proteins critical for motility and energy production [147]. Excessive carbonylation targets structural and metabolic proteins essential for motility. Cryopreservation is well known to induce ROS overproduction and lipid peroxidation [148]. It has been demonstrated that post-thaw sperm exhibit elevated carbonylation of key structural and metabolic proteins, including outer dense fiber protein 2 (ODF2), glutathione S-transferase (GST), NADH dehydrogenase, Ropporin, and triosephosphate isomerase [143]. These modifications impair sperm motility and ATP production. In addition, carbonylation affects signaling proteins critical for capacitation and acrosomal exocytosis; oxidized EF-hand calcium-binding proteins and α-SNAP, involved in vesicle fusion, have been detected in stressed spermatozoa, suggesting disruptions in Ca^2+^ signaling [149]. The modification of Hsp70 and histone-binding proteins suggests an additional role for carbonylation in protein misfolding and chromatin instability, which could impair DNA integrity and reduce fertilization potential [149]. Studies have shown that patients with asthenozoospermia have higher protein carbonylation levels, negatively correlating with sperm motility, fertilization rates, and embryo cleavage in intracytoplasmic sperm injection (ICSI) cycles [150].

Parallel to protein carbonylation, extensive lipid peroxidation (LPO) also compromises sperm functionality. While these aldehydes predominantly contribute to cellular damage, low levels of 4-hydroxynonenal may function as a signaling molecule [151]. Lipid peroxidation stiffens the flagellar membrane and disrupts lipid raft organization required for capacitation, impairing sperm motility and signaling. High lipid peroxidation levels in the acrosomal membranes have been shown to completely inhibit the release of hydrolytic enzymes necessary for zona pellucida penetration, effectively compromising the acrosome reaction [152,153]. In addition to protein damage via carbonylation, lipid peroxidation byproducts such as malondialdehyde and 4-hydroxynonenal can directly crosslink with DNA, causing fragmentation and chromatin decondensation [154]. Studies indicate that higher lipid peroxidation levels are associated with increased DNA fragmentation index, lower fertilization rates, and impaired embryonic development in assisted reproductive technologies [155].

Age-related oxidative stress is linked to declining sperm quality, with higher levels of lipid peroxidation and increased DNA fragmentation observed in aged bulls [156]. Together, protein carbonylation and membrane lipid peroxidation constitute interconnected arms of oxidative damage that dismantle spermatozoa’s structural and functional machinery, leading to reduced fertilization potential under oxidative stress conditions. Studies on bull and equine sperm have demonstrated that increased lipid peroxidation correlates with reduced motility and plasma membrane damage [156,157]. Lipid peroxidation is also a major contributor to oxidative stress damage during cryopreservation, affecting mitochondrial membrane potential and leading to ATP depletion and increased sperm apoptosis [158]. Cryopreserved spermatozoa exhibit elevated reactive oxygen species levels, exacerbating oxidative stress-induced apoptosis, but this effect can be mitigated by antioxidants such as selenium and L-carnitine [36,159,160,161,162].

To mitigate the propagation of lipid peroxidation and its downstream consequences, spermatozoa rely on intrinsic antioxidant defenses [23]. While seminal plasma provides a range of enzymatic and non-enzymatic antioxidants, within sperm cells, peroxiredoxins (PRDXs) constitute the primary endogenous defense system [157]. PRDXs are thiol-based peroxidases that catalytically reduce hydrogen peroxide and lipid hydroperoxides, curbing the initiation and amplification of lipid peroxidation [163]. Among these, peroxiredoxin 6 (PRDX6) plays a dual protective role: it detoxifies lipid peroxides and, through its calcium-independent phospholipase A_2_ (iPLA_2_) and lysophosphatidylcholine acyltransferase (LPCAT) activities, actively repairs oxidized phospholipids by excising damaged fatty acids and restoring membrane integrity [54,70]. This membrane repair function is critical for maintaining sperm motility and fertilization potential following oxidative insult. During physiological capacitation, a transient and reversible oxidation of PRDXs allows a regulated ROS surge necessary for redox-dependent signaling events, including tyrosine phosphorylation [98]. Subsequently, PRDXs are regenerated by the thioredoxin system, restoring antioxidant capacity and preventing the progression to oxidative stress. However, when PRDXs are overwhelmed or irreversibly inactivated, ROS accumulation leads to unchecked lipid peroxidation and secondary protein carbonylation, culminating in severe structural and functional sperm damage.

Experimental studies have demonstrated that inhibition or genetic deletion of PRDXs in sperm results in widespread oxidative lesions, impaired capacitation-associated signaling, loss of motility, and infertility [163,164]. Thus, PRDXs are indispensable for maintaining redox homeostasis in spermatozoa, ensuring that ROS serve as transient modulators of signaling rather than as agents of irreversible damage.

## 7. Protein S-Sulfhydration

Coenzyme hydrogen sulfide (H_2_S) is traditionally considered an environmental pollutant with a characteristic foul odor, often released from industrial activities and livestock waste [165]. However, in recent years, H_2_S has emerged as a critical endogenous signaling molecule in various physiological processes, including redox regulation [166]. One of its key mechanisms of action is through sulfhydration (persulfidation), a post-translational modification in which a cysteine thiol (-SH) group in a protein is converted into a persulfide group (-SSH) [167]. This reversible redox modification protects proteins from hyperoxidation, modulates enzymatic activity, protein-protein interactions, and subcellular localization. It has been implicated in cellular processes ranging from energy metabolism and immune response to cellular stress adaptation and gene expression. In metabolic tissues, for instance, it regulates insulin signaling [168] and mitochondrial bioenergetics [169], while in immune cells, it fine-tunes inflammatory cascades [170].

Hydrogen sulfide is enzymatically synthesized in mammalian tissues through three principal enzymes: cystathionine β-synthase (CBS), cystathionine γ-lyase (CTH), and 3-mercaptopyruvate sulfurtransferase (MPST), often in conjunction with cysteine aminotransferase (CAT) [171]. Spermatozoa from several mammalian species, including mice [172,173] and boar [174], possess the enzymatic machinery to generate H_2_S endogenously. In human spermatozoa specifically, a recent study has confirmed the presence of CBS, CSE, and MPST [175], where they are distributed along the sperm flagellum, with the strongest H_2_S production and sulfhydration activity localized in the midpiece, where mitochondrial metabolism is concentrated. The expression of these enzymes was found to be highest in immature sperm from the caput epididymis and gradually decreases through maturation in the cauda and during capacitation. Despite this reduction, H_2_S production remained detectable, indicating a functional role in sperm physiology.

Sulfhydration maintains sperm viability and may play a role in motility, energy metabolism and capacitation. Key proteins such as AKAP, GAPDH, tubulin, and heat shock proteins were identified as persulfidated targets [175]. Notably, these proteins are also known to undergo S-nitrosylation. This points to potential crosstalk between redox post-translational modifications, with sulfhydration possibly replacing or modulating nitrosylated states to fine-tune protein function during sperm activation. Kadlec et al. (2022) recently provided experimental evidence of this potential crosstalk in boar spermatozoa during oxidative stress conditions [176]. They found that adding NO (via SNP) and H_2_S (via NaHS) to the incubation medium significantly improved progressive motility and plasma membrane integrity more than either donor alone, indicating a synergistic protective effect under oxidative stress conditions. The antioxidant effect of H_2_S has been the subject of much research, with evidence that sulfhydration protects sperm from hyperoxidation during cryopreservation, acrylamide-induced toxicity, and cisplatin [148,177,178,179]. These findings are clinically relevant in the etiology of infertility since reduced H_2_S levels have also been observed in men with azoospermia and oligospermia [180].

The protective effects of H_2_S under oxidative stress appear to be dose- and donor-dependent. Slow-releasing H_2_S donors, such as GYY4137, have been shown to reduce oxidative damage and preserve mitochondrial function, thereby maintaining sperm motility. In contrast, high concentrations of fast-releasing donors like Na_2_S can lead to excessive H_2_S exposure, resulting in mitochondrial dysfunction and complete sperm immobility [174]. This pattern aligns with findings from Zhao et al. (2016), who demonstrated that exposure to high doses of Na_2_S, mimicking environmental insults, significantly impaired sperm motility through oxidative stress and disruption of energy-regulating pathways [181]. The collective findings on hydrogen sulfide (H_2_S) in sperm biology suggest that sulfhydration (S-sulfhydration) plays a key role in spermatogenesis and sperm maturation. This process is supported by endogenous H_2_S production, which declines as sperm mature and exit the testis due to reduced enzyme expression [175]. However, its role beyond sperm maturation remains indirect, as no studies have directly linked sulfhydration to capacitation or fertilization. For instance, it may influence capacitation-associated tyrosine phosphorylation since sulfhydration inactivates tyrosine phosphatases in insulin signaling pathways [168].

## 8. Methionine Oxidation

**Figure 8:**
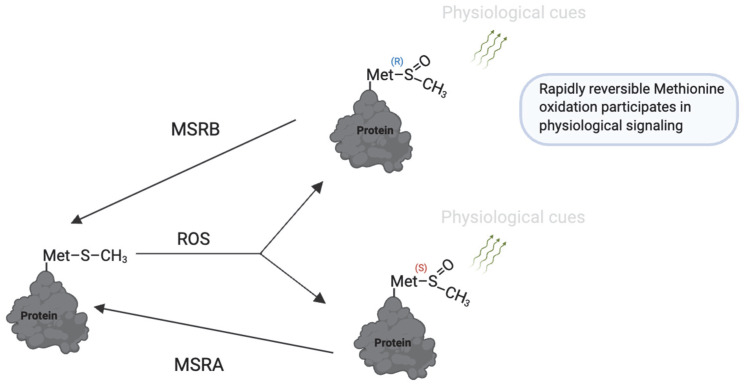
Reversible methionine oxidation as a redox regulatory mechanism. Under oxidative conditions, methionine (Met) is converted to methionine sulfoxide (MetO), which exists in two stereoisomeric forms: Met-S-O and Met-R-O. These modifications can regulate protein function, folding, and stability. The process is reversed by methionine sulfoxide reductases (MsrA and MsrB), which specifically reduce the S- and R-isomers, respectively, restoring methionine and allowing dynamic redox cycling. This reversible system is a redox switch that protects methionine residues from irreversible damage and contributes to fine-tuning redox-sensitive protein activity during physiological processes. https://app.biorender.com/illustrations/681527a353b88e93b9976da4. Assessed on 3 June 2025.

Oxidation may also occur selectively at methionine residues in proteins, although less frequently than cysteine oxidation [182]. Rosenfeld et al. (2021) [183] and Aledo (2019) [182] recently provided comprehensive reviews on the mechanisms and biological roles of methionine oxidation. Upon exposure to oxidants such as hydrogen peroxide (H_2_O_2_) and hypochlorous acid (HOCl), methionine residues are oxidized to methionine sulfoxide (MetO). This reaction can be catalyzed by the MICAL family of monooxygenases [184]. The oxidation of MetO is reversible and stereospecific, catalyzed by two distinct enzymes: methionine sulfoxide reductase A (MSR-A), which reduces the S-stereoisomer, and methionine sulfoxide reductase B (MSR-B), which reduces the R-stereoisomer [185] (Figure 8).

In addition to its established structural role, methionine oxidation contributes to functional regulation and antioxidant protection [182]. The antioxidant role of methionine is evident from studies demonstrating that proteins rich in methionine residues, such as ⍺ -macroglobulin, can tolerate extensive oxidation without loss of activity, acting as ROS sinks to protect other less protected proteins [182,186]. Furthermore, reversible oxidation of methionine residues influences the redox regulation of actin polymerization, reducing polymerization efficiency and affecting filament stability [187,188]. The reversibility of these modifications by MRSA/B restores polymerization potential, allowing dynamic regulation of cytoskeletal organization in response to redox signals [189]. Additionally, calmodulin, a protein lacking cysteine residues but enriched in methionine, exhibits decreased sensitivity to calcium ions upon methionine oxidation, suggesting a potential mechanism for redox-based regulation of its activity [183,190].

Methionine oxidation notably occurs preferentially near phosphorylation sites, suggesting interactions between oxidation and phosphorylation signaling pathways [191]. Several proteins and physiological processes have been proposed to be regulated by the MSR system, including alpha-1-antitrypsin (α1-AT), high-density lipoproteins (HDLs), inhibitor of kappa B-alpha (IκBα), potassium channels, thrombomodulin (TM), tissue plasminogen activator (t-PA), lipoxygenases, and heat shock proteins (Hsps) [192].

Despite growing recognition of the functional significance of methionine oxidation, its role in sperm physiology remains poorly characterized, with limited and conflicting findings. For instance, studies report beneficial outcomes of methionine supplementation in sperm cryopreservation media, particularly enhancing semen quality in buffalo spermatozoa, which express high levels of MSRA [193]. Similar elevated MSRA expression is documented in mouse spermatozoa [194]. However, other research demonstrates improvements in sperm concentration and motility upon dietary methionine restriction in mice, indicating potentially detrimental effects of methionine [195]. Given the critical role of redox signaling in sperm function, methionine oxidation likely serves as an essential modulator of sperm physiology. Therefore, further targeted studies are warranted to clarify the specific molecular mechanisms of methionine oxidation, its interplay with other redox events, and its overall impact on sperm function.

## 9. Conclusions

RONS play a dual role in spermatozoa: at mild (low) physiological levels, they initiate essential redox signaling events required for fertilization, while at elevated levels, they induce oxidative stress that impairs fertility. Despite their importance, thresholds separating physiological from pathological oxidation remain technically elusive, making it necessary to rely on redox-induced protein modifications as biomarkers of levels of RONS exposure. Reversible modifications such as S-sulfenylation, S-nitrosylation, S-sulfhydration, and methionine oxidation generally reflect low ROS levels, whereas irreversible ones like sulfonic acid formation, tyrosine nitration, and protein carbonylation mark oxidative damage. Notably, modifications like S-glutathionylation, S-CoAlation, and S-sulfinylation exist in a gray zone; reversible under physiological conditions but reflecting pathology when persistent. Among these, S-CoAlation has emerged as a particularly promising candidate for assessing human sperm quality, given the universal abundance of CoASH in all cell types, redox sensitivity, and emerging roles in capacitation and oxidative stress protection. However, critical gaps remain. For instance, the redox mechanisms underlying non-cysteine modifications such as methionine oxidation are underexplored, and the identity of enzymes responsible for reversing modifications like S-CoAlation, (a putative “CoA-redoxin”) remains unknown. Given the high prevalence of oxidative stress in male infertility, future research should prioritize mapping the protein targets and regulatory consequences of redox modifications in spermatozoa. Translationally, these insights could support the development of redox-based diagnostics and therapeutics tailored to individual oxidative profiles. For example, pharmacological agents mimicking or enhancing endogenous reducing systems might restore sperm function in men with redox dysregulation. Addressing these unanswered questions will not only clarify the redox landscape of sperm physiology but also advance precision medicine approaches in male reproductive health.

## Figures and Tables

**Table 1:** Effects of S-Nitrosylation and Tyrosine Nitration on Sperm Function.

Modification	Effect	Species	Reference
S-Nitrosylation	Enhances capacitation	Human, Boar	[64,66]
	Enhances cryosurvival	Human, Rooster	[60,61,67]
	Enhances motility	Human, Ram	[68,69]
Tyrosine Nitration	Impairs motility	Boar, Human	[72,74,75]
	Impairs acrosome reaction	Human	[35,76]
	Promotes mitochondrial permeability transition (MPT)-driven apoptosis	Human	[77]

**Table 2:** Functional Roles of Protein S-CoAlation in Various Biological Systems.

Effect of S-CoAlation	Model	Reference
Antioxidant function	Mammalian cells, Bacteria, Amoeba	[127,135,136]
CoAlation induced by carbon starvation, sporulation and oxidative stress	Bacillus megaterium, Bacillus subtilis	[135]
Reversible inactivation of metabolic enzymes (e.g., GAPDH) under oxidative stress	Staphylococcus aureus	[137]
Reversible inactivation of antioxidant enzymes (e.g., Peroxiredoxin 5)	Mammalian Cardiomyocytes	[139]
Modulates signaling pathways by reversible inhibition of kinases (e.g., S6K1, Aurora kinase A)	Mammalian cells (HEK293)	[129,133]
Inhibition of catalytic activity through ADP moiety interaction (e.g., NME1/NME2)	Mammalian cells (HEK293)/Recombinant proteins from *E. coli* (BL21 DE3)	[130,131]
Increases in human spermatozoa exposed to oxidative stress, suggesting antioxidant function	Human spermatozoa	[140]
Decreases during capacitation, possibly relieving inhibition of phosphorylation pathways	Human spermatozoa	[140]

## Abbreviations

The following abbreviations are used in this manuscript:

ROS	Reactive Oxygen Species
RNS	Reactive Nitrogen Species
H_2_O_2_	Hydrogen Peroxide
NO	Nitric Oxide
ONOO^−^	Peroxynitrite
PUFA	Polyunsaturated Fatty Acid
MDA	Malondialdehyde
4-HNE	4-Hydroxynonenal
PRDX	Peroxiredoxin
PRDX6	Peroxiredoxin 6
PKA	Protein Kinase A
PKC	Protein Kinase C
GPX	Glutathione Peroxidase
GST	Glutathione-S-Transferase
TXNRD	Thioredoxin Reductase
Trx	Thioredoxin
GSH	Reduced Glutathione
GSSG	Oxidized Glutathione
MSR	Methionine Sulfoxide Reductase
MSRA/B	Methionine Sulfoxide Reductase A/B
SNO	S-Nitrosylation (S-NO-modified proteins)
SSG	S-Glutathionylation (S-SG-modified proteins)
SSC0A	S-CoAlation (Coenzyme A-modified proteins)
SOH	Sulfenic Acid Modification
SO_2_H	Sulfinic Acid Modification
SO_3_H	Sulfonic Acid Modification
SSH	Sulfhydration (Persulfidation)
ZP	Zona Pellucida
EF-hand proteins	Calcium-binding proteins (named for their helix-loop-helix structure)
Hsp70	Heat Shock Protein 70
S6K1	Ribosomal Protein S6 Kinase 1
ODF2	Outer Dense Fiber Protein 2
GAPDHS	Glyceraldehyde-3-Phosphate Dehydrogenase, Sperm-specific
AKAP	A-Kinase Anchoring Protein
RyR	Ryanodine Receptor
SNAP	Soluble NSF Attachment Protein
iPLA_2_	Calcium-Independent Phospholipase A_2_
LPCAT	Lysophosphatidylcholine Acyltransferase
